# Arterial Versus Venous Blood Gas Analysis Comparisons, Appropriateness, and Alternatives in Different Acid/Base Clinical Settings: A Systematic Review

**DOI:** 10.7759/cureus.41707

**Published:** 2023-07-11

**Authors:** Lillian Saberian, Maria Sharif, Mehdi Aarabi, Behrooz Broumand, Mohammad A Shafiee

**Affiliations:** 1 Medical Science, University of Toronto, Toronto, CAN; 2 Medicine, Shahrekord University of Medical Sciences, Shahrekord, IRN; 3 Medicine, University of Toronto, Toronto, CAN; 4 University Health Network, Toronto General Hospital, Toronto, CAN; 5 Nephrology, Pars Hospital, Tehran, IRN

**Keywords:** gas analysis, central, peripheral, comparison, venous blood gas, arterial blood gas

## Abstract

Arterial blood gases (ABGs) are routinely done in critical clinical settings to ascertain acid-base status. Due to difficulties and the potential side effects following arterial blood sampling, much research has been done to find the possibility of using venous samples as an alternative. However, this comparison needs to be evaluated in various contexts. Hence, this systematic review aims to explore the differences, appropriateness, and alternatives of arterial versus venous blood gas (VBG) analysis in different acid-base states. A comprehensive literature search was conducted through electronic databases using the terms “ABG,” “VBG,” “Arterial Blood Gas,” “Venous Blood Gas,” and “Gas analysis.” Studies' qualities were assessed by using Newcastle - Ottawa Quality Assessment Scale. Of 531 articles, 22 were included in the study after title, abstract, and full-text screening. Based on the Newcastle - Ottawa Quality Assessment Scale, 23% of the studies had good quality (score ≥ 7), 77% fair quality (score 2-6), and none of the studies had poor quality (score ≤ 1). Moreover, 22.5% of the included articles found a strong correlation between ABG and VBG. 73% compared arterial and VBG parameters among patients with any clinical contexts, 22.5% in respiratory diseases, and 4.5% in metabolic conditions, and their results had a significant disparity. There was a considerable discrepancy among authors about the appropriateness and utilization of VBG as an alternative to ABG. Our findings suggest that those studies did not consider physiological differences between venous and arterial blood values and obviated the significance of sampling procedures.

## Introduction and background

A common belief is that arterial blood gases (ABGs) provide crucial information about critically ill patients' acid-base status, oxygenation, and electrolyte levels. However, the investigation is invasive and causes pain and discomfort to the patient. In addition, complications such as severe bleeding, hematoma, thrombosis, or infection can arise. The more commonly used venous blood sample is easier to obtain and has fewer complications. For this reason, many studies have been conducted to compare the agreement between venous and ABG values and assess their accuracy to evaluate whether venous samples can be used as an alternative to arterial.

Considering the physiology of blood circulation, capillaries drain the tissue and provide information about the interstitial environment. When evaluating metabolic derangements, capillary samples give a close approximation of events occurring at the cell level. Obtaining capillary samples are impractical; therefore, venous samples can be used as they are the closest to the capillary system. Hence, in some metabolic conditions, venous gas measurements could provide better information about the events at the cell levels than arterial samples [1].

When choosing a venous sample over an arterial, besides patients' clinical status, it is essential to consider factors affecting blood circulation, such as sampling site, puncture site temperature, and blood flow rate [1-5]. This systematic review aimed to provide an overview of the appropriateness, choices, and alternatives of arterial versus venous blood gas (VBG) analysis in different acid-base states.

## Review

Material and methods

Literature Search

A comprehensive literature search was conducted from January to February 2023 using electronic databases, Embase, MEDLINE, EBM Reviews, Cochrane DSR, DARE, and Health Technology Assessment via Ovid, and CINAHL via EBSCO. The search terms used were “Arterial Blood Gas,” “Venous Blood Gas,” “ABG,” “VBG,” and “Gas analysis.” The aim was to explore the correlation between arterial and VBG measurements. Relevant articles were included by applying Medline's MeSH terms adjusted for the database mentioned above. The protocol was registered in PROSPERO (CRD42023436918).

Inclusion and Exclusion Criteria

All studies included prospective and historical cohorts, cross-sectional, or case-control studies that compared VBGs (peripheral or central) with ABGs among adult patients, were included. We included studies reporting the mean difference and standard deviation for one or more data, including the potential of hydrogen (pH), partial pressure of carbon dioxide (pCO_2_), bicarbonate (HCO_3_), and partial pressure of oxygen (pO_2_), for paired, sequentially obtained peripheral or central venous and arterial blood samples. Studies in which venous blood samples were obtained centrally or during cardiopulmonary bypass were also included. 

Non-English articles, study protocols, experimental designs with or without a control group, reviews, and conference abstracts were excluded. Relevant abstracts were considered for full-text review, and eligible studies were included for data extraction.

Study Population

Studies comparing VBGs (peripheral or central) to ABGs among adult patients aged ≥ 18 who were hospitalized in the emergency department (ED) or intensive care unit (ICU) due to respiratory, metabolic, or both diseases were included. Studies with animal populations or pregnant subjects were excluded. 

Data Extraction

The first and second authors independently conducted title and abstract screenings, as well as full-text reviews. Any disagreements were resolved through discussions with the corresponding author. Data from the included study are stored in Excel Spreadsheet as the first author's name, publication year, type and setting of the study, sample size, mean age, related diseases, pH, pCO_2_, HCO_3_, and pO_2_. Information about ABG-VBG means differences regarding pH, pCO_2_, HCO_3_, and pO_2_ values from all included articles were extracted and, if necessary, calculated. 

Quality Assessment

The Newcastle-Ottawa Quality Assessment Scale was used to assess the quality of the cohort and case-control studies [6], and its adapted version was utilized for the cross-sectional studies [7]. This scale is categorized into selection, comparability, and outcome or exposure for cohort or case-control studies. The quality of each study was classified as “good” (score ≥7 points), “fair” (2-6), and “poor” (score ≤1). Studies with fair to good quality were included to improve the validity of this systematic review. Two reviewers with expertise in quality assessment tools conducted the initial quality assessment of the articles independently. Following this, an independent statistician performed a re-evaluation of the quality assessment. In the case of any disagreements, they were resolved through discussions with the corresponding author, who is an expert in this field.

Results

**Figure 1:**
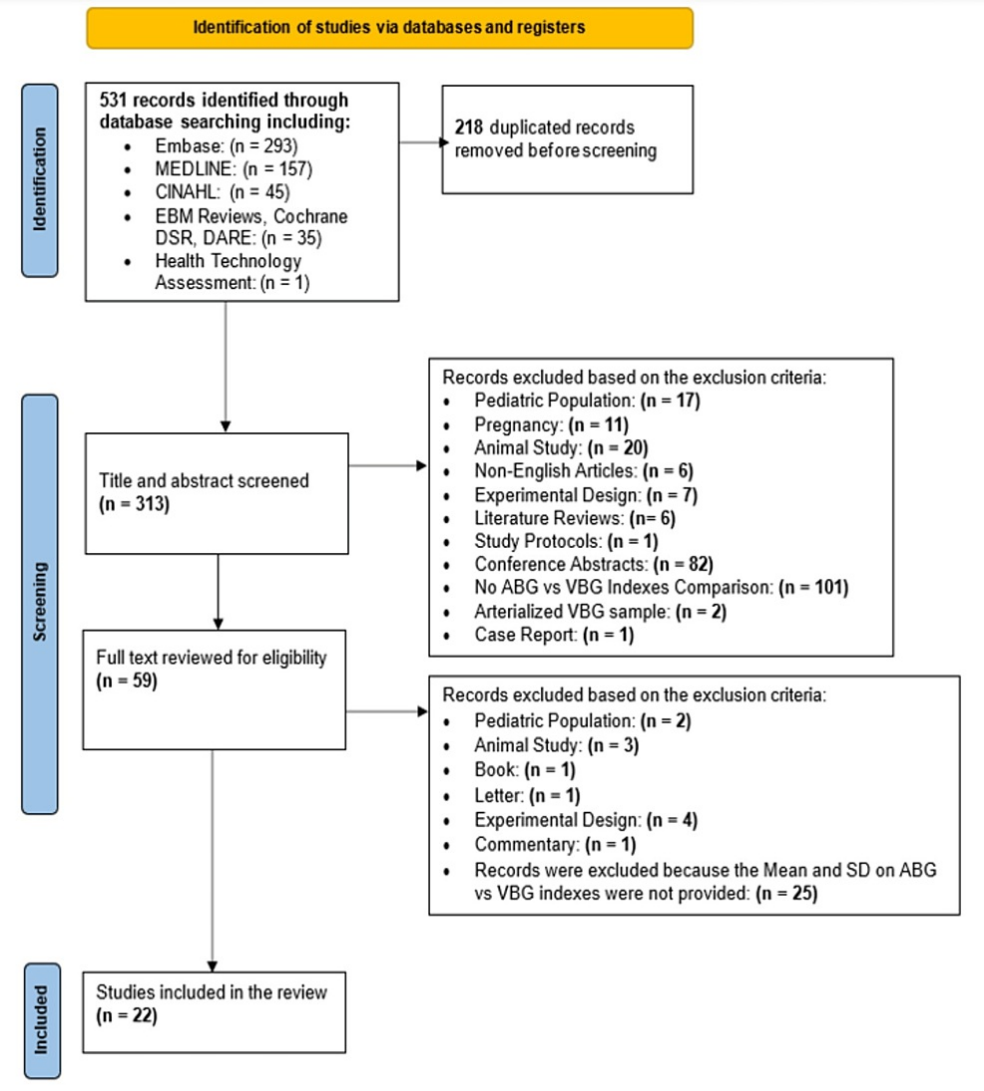
PRISMA flowchart for the article selection process

Five hundred thirty-one articles were retrieved and subjected to title, abstract, and full-text screening. Finally, 22 articles were included in this systematic review. Preferred reporting items for systematic reviews and meta-analyses (PRISMA) flowchart shows the article selection process (Figure 1).

Quality Assessment Results

Among 22 articles, 18 were cross-sectional, three were cohort, and one was case control. Based on the Newcastle-Ottawa Quality Assessment Scale, 23% of the studies had good quality, 77% fair quality, and none had poor quality (Table 1).

**Table 1:** The percentage of articles based on the quality assessment results (N=22) Values are expressed in percentages (%), N: Total number of articles Good quality: score ≥ 7 points; Fair quality: score = 2-6; Poor quality: score ≤ 1

Quality Score	Cohort	Cross-sectional	Case-control	Total
Good Quality	9	14	0	23
Fair Quality	4.5	68	4.5	77
Poor Quality	0	0	0	0

Main Outcomes

Fourteen studies used peripheral VBG (PVBG), three used central VBG (CVBG), two used PVGB and CVBG samples, and three articles did not specify the puncture site. Among included papers, 22.5% found a strong correlation between arterial and venous parameters and recommended using PVBG or CVBG instead of ABG, and 55% saw a moderate correlation between ABG and VBG (CVBG or PVBG). They showed that VBG might be a good substitute for ABG, as long as using VBG and pulse oximetry (SpO_2_), CVBG and PVBG simultaneously, using VBG in conjunction with a correction factor, or using VBG for trending purposes (Table 2).

**Table 2:** The percentage of articles based on their findings about the strength of correlation between ABG and VBG in different clinical conditions (N=22) Respiratory conditions: Diseases or disorders which disrupt the respiratory system. These conditions include chronic obstructive pulmonary disease (COPD), asthma, pneumonia, and pulmonary embolism. Metabolic conditions: Diseases or disorders which disrupt normal metabolism, and they include diabetes mellitus, diabetic ketoacidosis, renal failure, and sepsis. Values are expressed in percentages (%). N: Total number of articles; ABG: Arterial blood gas; VBG: Venous blood gas; CVBG: Central venous blood gas; PVBG: Peripheral venous blood gas

Degree of correlation	Any clinical conditions				Respiratory conditions				Metabolic conditions	Total (%) (based on the degree of correlation)
	CVBG	PVBG	CVBG & PVBG	Not specified	CVBG	PVBG	CVBG & PVBG	Not specified	PVBG	
Strong correlation	0	4.5	4.5	0	0	9.1	0	0	4.5	22.5
Moderate correlation	9.1	23	4.5	9.1	0	9.1	0	0	0	55
Poor or no correlation	4.5	9.1	0	4.5	0	4.5	0	0	0	22.5
Total (%) (based on the clinical condition)	73				22.5				4.5	100

In the studies among patients with any clinical context, 9% found a strong agreement between some arterial and venous parameters and recommended using VBG (PVBG or CVBG) instead of ABG in critically ill patients. From five studies comparing ABG and PVBG among patients with respiratory conditions [4,8-11], 9.1% found a strong correlation between ABG and VBG values of pH, pCO_2_, and HCO_3_ [4,8]. Finally, one study compared ABG and PVBG among patients with metabolic disorders (Table 2). Their results showed that a venous blood sample could evaluate the acid-base status in uremic and diabetic ketoacidosis (DKA) patients [12].

Secondary Outcomes

Table 3 represents the percentage of articles based on their findings regarding the correlation strength between arterial and venous parameters, including pH, pCO_2_, HCO_3_, and pO_2_.

**Table 3:** The percentage of articles based on their findings about the correlation strength among ABG and VBG components (pH, pCO2, HCO3, pO2) (N=22). Values are expressed in percentages (%). N: Total number of articles; ABG: Arterial blood gas; VBG: Venous blood gas; pH: Potential of hydrogen; pCO_2_: Partial pressure of carbon dioxide; HCO_3_: Bicarbonate; pO_2_: Partial pressure of oxygen

Degree of Correlation	pH	pCO_2_	HCO_3_	pO_2_
Strong Correlation	63.5	63.5	59	13.5
Moderate Correlation	14	9	9	4.5
Poor or no Correlation	18	23	14	32
Not Specified	4.5	4.5	18	50

Discussion

ABGs are routinely done in critical clinical settings, especially in the ED, to ascertain the adequacy of ventilation and oxygenation, acid-base status, and electrolyte levels. Due to the difficulty of obtaining arterial sampling and its potential adverse effects, many studies have been conducted to evaluate the possibility of using venous blood sampling as an alternative. The findings of this systematic review indicated that some studies did not consider physiological differences between the blood circulation in peripheral venous when compared with arterial blood values in clinical decision-making. Some obviated clear indications of the anatomical site, temperature differences, and sampling methods, which could impact blood circulation.

Phillips and Peretz [13] assessed 41 critically ill patients and found that CVBG is an accurate substitute for ABG analysis in metabolic status and deciding on further management. Likewise, Gokel et al. [12] assessed metabolic disorders and represented using PVBG as an alternative to ABG in uremic or DKA patients. They supported using venous blood samples to evaluate the acid-base status in uremic and DKA patients instead of ABG samples; however, they did not use CVBG sampling in their assessment [12].

Regarding respiratory disturbances, four studies did not recommend estimating arterial pO_2_ from VBG values in exacerbations of COPD (ECOPD) [4,8,9,11]. Some also suggested using pulse oximetry with VBG analysis to estimate the blood gas values in assessing respiratory conditions [8,10,11]. Kozaci et al. [10] found that if O_2_ saturation is less than 90% in patients with respiratory alkalosis, VBG could be a substitute for estimating ABG's pH, pCO_2_, HCO_3_, and pO_2_ values.

Among studies on patients with any clinical contexts (metabolic, respiratory, or complex acid-base disorders), a considerable heterogenicity in the results was seen [13-28]. Bohloli [16] suggested using single regression prediction models to predict arterial PCO_2_ and HCO_3_, to replace ABG with PVBG. Ibrahim et al. [17] did not recommend using PVBG as an alternative to ABG because they found substantial variability in the arteriovenous difference for pCO_2_ values. Also, Razi et al. [18] concluded that VBG could not be an alternative for ABG in mechanically ventilated patients since PVBG parameters had no close correlation with ABG values.

On the other hand, five studies found that PVBG might be a reliable substitute for ABG [19-23]. They recommended using PVBG and pulse oximetry [21], PVBG for trending purposes or in conjunction with a correction factor [23] or using PVBG in the initial assessment of adult patients in the ED [22].

Kim et al. [19] represented that peripheral venous pH, pCO_2_, HCO_3_, and total CO_2_ might be used as a substitute for their arterial equivalents. However, they underrepresented cardiogenic or hypovolemic shock and the anatomical site of obtaining PVBG sampling, resulting in a different correlation between arterial and venous values. The anatomical location of blood sampling reflects the metabolism of tissues from which the sample has been obtained. More metabolically active organs have higher venous drainage than those less active. They also did not compare and specify arterial and venous values of pO_2_ in their study.

Treger et al. [24] and Zeserson et al. [25] compared ABG with PVBG and CVBG samples of patients with any clinical context admitted to the ICU or ED. They also did not recommend using CVBG or PVBG to estimate pO_2_ values as a substitute for ABG [24,25].

One study conducted on patients undergoing mechanical ventilation after open-heart surgery in the ICU revealed that central venous saturation O_2_ did not consistently predict arterial saturation O_2_. Nonetheless, the study authors suggested substituting ABG analysis with CVBG analysis in specific situations [15]. Malinoski et al. [14] compared ABG and CVBG values of pH, pCO_2_, and base excess but not those of HCO_3_ and pO_2_ in mechanically ventilated trauma patients admitted to ICU. They represented that CVBG could not be substituted for ABG during the initial resuscitation phases.

Chung et al. [26], in their studies on diverse populations in a trauma center, found that VBG was not an accurate alternative for ABG because of poor agreement in acidemia and hypercarbia. While two other studies [27,28] on patients with different medical conditions in the ED recommended substituting ABG with VBG for measuring pH and HCO_3_ but not for pCO_2_ and pO_2_. However, these studies did not specify whether venous blood samples were obtained centrally or peripherally, which could affect the variability of the VBG values.

In comparing ABGs and VBGs, essential factors should be considered to provide accurate and valid results. The primary factor is considering the physiological differences between arterial and PVBG parameters. For example, the PVBG has considerably less O_2_, more CO_2_, and a lower pH than the arterial one because of the high O_2_ utilization by the organs distal to the peripheral venous blood. Moreover, differences in patients' tissue perfusion affect the speed of venous return and its consequences on the O_2_ and CO_2_ tensions. CO_2_ in PVBG will be lower than ABG in high venous returns like vasodilation, while in low venous returns like cardiac failure, it will be higher than ABG [2].

A CVBG represents the venous return and the amount of oxygen residue from organs supplied by the central venous catheter location. As a result, CVBG may not correlate with venous oxygen saturation (SvO_2_) during shock or in unstable patients with severe acid-base disturbances, in which arterial confirmation with ABG is recommended [29]. In CVBG, gases may also be impacted if a patient has a blood flow problem like ischemia or air aspiration into the syringe during sampling. Therefore, the origin of a venous sample for blood gas analysis, whether it was drawn from a peripheral stab or a central venous catheter, should be considered and documented in the study.

Furthermore, the temperature of organs for obtaining the peripheral venous blood sampling is essential in deciding when to substitute a PVBG for ABG since tissue perfusion depends on organ temperature [5]. Moreover, the PVBG sample should be analyzed immediately after the collection or cooled to 5°C to avoid spoiling its components and inaccuracy [30].

Another essential factor is considering the clinical status of the patients in deciding when to use VBG as the alternative to ABG. For instance, in metabolic disturbances, blood samples taken from the central venous are more practical and accurate than ABG in determining metabolic events happening at the cell level [1]. Conversely, ABG is the choice in respiratory conditions because ABG is an indicator of events at the lung level [3,4]. However, arterialization, pre-warming the puncture site to 45 to 50˚C using infrared light before collecting venous blood samples, closely reflects arterial PO_2_, O_2_ saturation, and PCO_2_ [5]. As a result, arterialized VBG can be used even in respiratory events; hence there is very good tissue perfusion at the sample site.

Limitations

Some limitations should be considered when interpreting the results of this systematic review. First, this study was not included non-English articles. Second, some critical information was unavailable from included studies, such as sampling methods and the puncture-site temperature. Finally, some papers did not report data about the P-value, standard deviation, and mean value of ABG-VBG, making comparing ABG and VBG challenging.

## Conclusions

Considerable heterogeneity and discrepancies have been observed among studies assessing the accuracy of VBG as an alternative to ABG analysis. These variations can be attributed to disregarding physiological differences between venous and arterial blood circulations, variations in the clinical status of patients, and inconsistencies in the sampling procedures.

While capillary samples directly reflect cellular-level changes, they pose challenges in obtaining them during clinical practice. In this regard, venous samples offer a closer approximation to capillary tissue and can provide valuable insights into metabolic derangements. For instance, jugular vein samples can offer crucial information regarding metabolic disturbances in brain cells. Hence, venous blood sampling is a superior alternative to ABG analysis for patients experiencing metabolic disturbances.

Conversely, arterial blood, driving directly from pulmonary circulation, accurately reflects respiratory conditions. Thus, ABG analysis remains the gold standard for critically ill patients receiving ventilator support or those with other respiratory conditions, providing comprehensive information about respiratory status and acid-base balance.

Furthermore, it is essential to note that CVBG is most accurate in assessing average metabolic disturbances of cells, whereas ABG is the preferred choice for evaluating respiratory conditions. However, in low tissue perfusion states, such as cardiogenic or hypovolemic shock, PVBG can reflect metabolic derangements in peripheral tissues but may not accurately represent ABG values due to differences in correlation between arterial and venous values.

Future studies of simultaneous ABG, mixed CVBG, and appropriate PVBG samples, with a clear indication of anatomical site, temperature differences, sampling method, or arterialized sample, would provide more insight into proper gas analysis for each category.
